# Continuous Vaginal Bleeding Induced By EGFR-TKI in Premenopausal Female Patients With EGFR Mutant NSCLC

**DOI:** 10.3389/fonc.2022.805538

**Published:** 2022-06-07

**Authors:** Min Yu, Xiaoyu Li, Xueqian Wu, Weiya Wang, Yanying Li, Yan Zhang, Shuang Zhang, Yongsheng Wang

**Affiliations:** ^1^ Department of Thoracic Oncology of Cancer Center, West China Hospital, Sichuan University, Chengdu, China; ^2^ Institute of Drug Clinical Trials, West China Hospital, Sichuan University, Chengdu, China; ^3^ Department of Pathology, West China Hospital, Sichuan University, Chengdu, China; ^4^ Department of Biotherapy, Cancer Center, West China Hospital, Sichuan University, Chengdu, China

**Keywords:** vaginal bleeding, non-small cell lung cancer, EGFR tyrosine kinase inhibitor, epidermal growth factor receptor, premenopausal female

## Abstract

EGFR-TKI is widely used for EGFR-mutant NSCLC patients. Bleeding is reported as a neglected adverse effect induced by EGFR-TKI. Female patients with lung adenocarcinoma have a high frequency of EGFR mutations. This study investigated the effect of EGFR-TKI on the menstrual cycle, especially on bleeding, in women of childbearing age. The underlying mechanism was further investigated in a patient with severe bleeding. We retrospectively investigated the effects on menstrual cycle in premenopausal female NSCLC patients who underwent EGFR-TKI treatment during 2013 to 2019. Menstrual changes including cycle disorders and prolonged bleeding were investigated *via* questionnaire survey. EGFR signaling, ER, PR and tissue factor expression were analyzed in endometrium tissue obtained from a 43-year-old patient who suffered from continuous vaginal bleeding during treatment with erlotinib and osimertinib. Among 42 premenopausal female patients taking EGFR tyrosine kinase inhibitor, 69.05% patients experienced abnormal menstruation. In women with abnormal menstruation, 41.37% had profuse menstruation and 20.69% had irregular menstruation. In most cases, the abnormal vaginal bleeding stopped when suspending EGFR-TKI. The EGFR-TKI induced abnormal vaginal bleeding might be associated with low progesterone level, decreased EGFR activation and tissue factor (TF) expression in endometrial tissues. EGFR-TKI unusually induce abnormal vaginal bleeding in premenopausal female NSCLC patients, which may be attributed to progesterone/EGFR/TF signaling. Megestrol acetate may be an available and effective drug for the uncommon adverse effect.

## Background

Epidermal growth factor receptor (EGFR) activation mutation has been demonstrated to be driver genes in non-small cell lung cancer (NSCLC) that can benefit from oral EGFR tyrosine kinase inhibitors (1, 2). Most of EGFR tyrosine kinase inhibitor (EGFR-TKI) have been developed to block autophosphorylation and subsequent signal transduction, including both wild type and mutant EGFR signaling (3, 4). Therefore, EGFR-TKI inevitably generate some adverse events, including skin toxicity, gastrointestinal reactions, and so on, owing to extensive EGFR expression in both tissue and normal tissue (5).

Clinically, some female patients complained of menstrual abnormalities, even prolonged or continuous bleeding. As known, EGFR mutation rate was significantly higher in women, especially in younger women, while these population may have normal menstruation (6). Previous studies have shown that EGF/EGFR signaling is closely related to growth, differentiation of human endometrium and even hemostasis in the menstrual cycle (7). Abnormal menstruation, especially vaginal bleeding, is a common but possibly severe gynecological symptom. In this situation, it is necessary to clarify whether EGFR-TKI induces the continuous bleeding of the menstrual cycle and its underlying mechanism for clinical treatment.

Herein, we investigated menstrual changes in premenopausal women after receiving EGFR-TKI treatment, in particular prolonged and increased bleeding, and explored potential mechanisms in a severe vaginal bleeding patient.

## Methods

### Patient and Clinical Data Collection

Premenopausal female patients with EGFR-mutant NSCLC and received EGFR-TKI during 2013 to 2019 were investigated whether there was an abnormal menstruation. Clinical characteristics and the following data were collected: bleeding pattern of the vaginal bleeding (duration, volume, frequency), types of EGFR inhibitor, applied medical interventions to stop the bleeding, and the subsequent outcome of the bleeding. The Ethics Committee of West China Hospital has exempted informed consent.

### Endometrial Sample Analysis

A 43-year-old patient who suffered from continuous vaginal bleeding and diagnostic curettage during treatment with erlotinib and osimertinib. Endometrial sample in the typical case was used for the examination of EGFR, p-EGFR and TF expression by immunohistochemistry. Two samples from non-cancer patients were used as controls.

## Results

### Patients and Clinical Data

**Figure 1:**
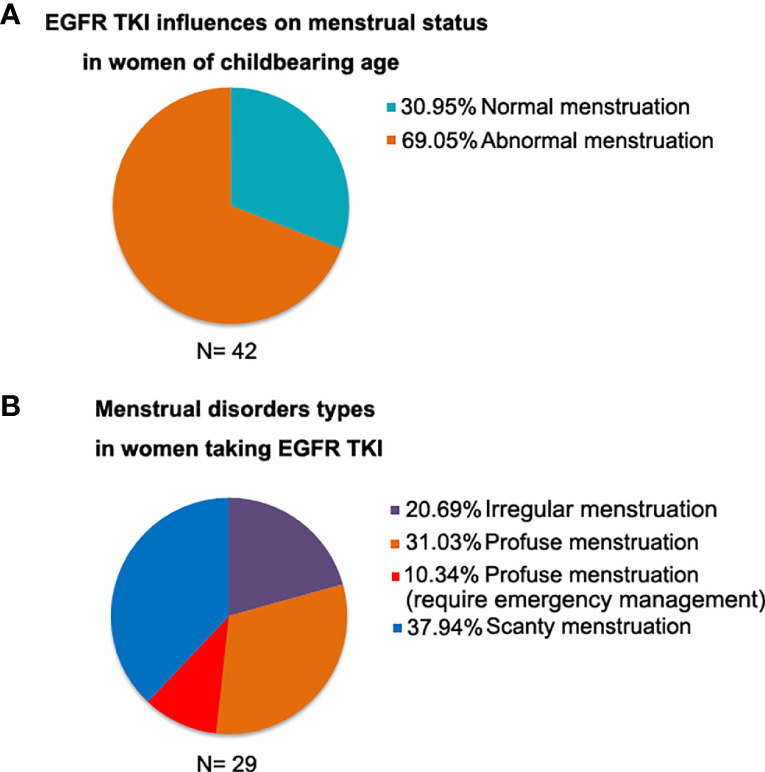
The menstrual status in 42 premenopausal women during EGFR-TKI treatment. **(A)** 69.05% women experienced abnormal menstruation; **(B)** Among women with abnormal menstruation, 41.37% had profuse menstruation.

We retrospectively analyzed the menstrual status in 42 premenopausal women during EGFR-TKI treatment. It indicated that 69.05% women experienced abnormal menstruation. Among women with abnormal menstruation, 20.69% women had irregular menstruation, 41.37% had profuse menstruation, and 37.94% had scanty menstruation (**Figure 1**).

### A Case Report and Case-Based Mechanism

**Figure 2:**
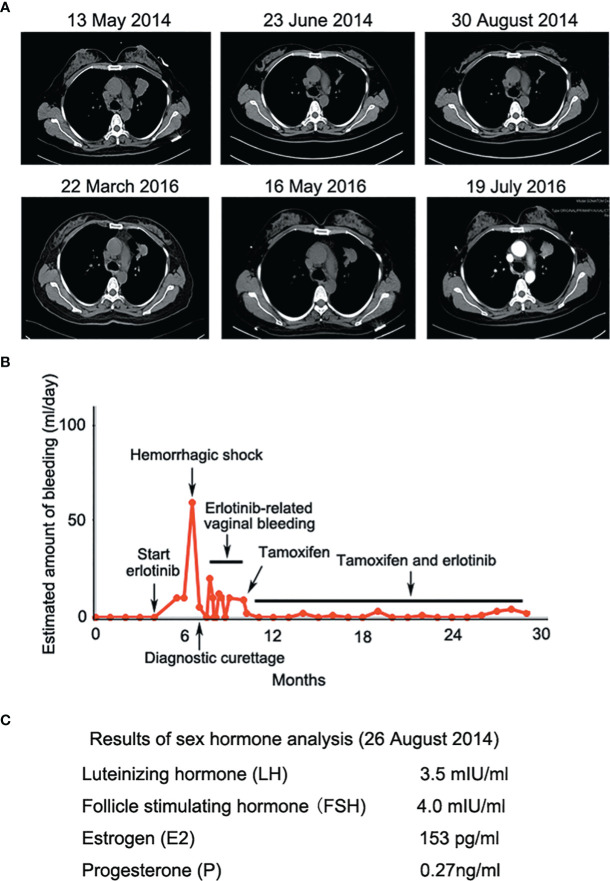
**(A)** Erlotinib decreased lesion in the upper lobe of the left lung significantly at the early stage of treatment; **(B)** Erlotinib-related vaginal bleeding during the treatment; **(C)** Sex hormone tests showed low levels of progesterone during luteal stages of the menstrual cycle.

A 43-year-old female Chinese never-smoker was admitted to local hospital in February 2014, for severe headache and vomiting. Radiological investigations showed intracranial space occupying lesion suggestive of metastasis. The patient underwent craniotomy and excision of the lesion. Histological examination and CT scan revealed a brain metastasis of lung adenocarcinoma. After 2 cycles of chemotherapy using docetaxel and nedaplatin, CT image suggested that adenocarcinoma of the left lung had increased dramatically. The patient was admitted to our hospital in May 2014. CT image showed a 4.0 cm diameter mass in the upper lobe of the left lung, with multiple nodules of different sizes in both lungs. Targeted next-generation sequencing (NGS) was performed on DNA extracted from the tumor biopsy specimen, revealing that the tumor harbored EGFR exon 19 deletion. The patient was treated with erlotinib, 150 mg daily from 18 May 2014. After 5 weeks of treatment, chest CT showed mass in the upper lobe of the left lung shrunk significantly (**Figure 2A**). Nevertheless, the patient complained of continuous vaginal bleeding for 2 weeks, with a volume of more than 10 ml daily. The hemorrhage worsened over time, and the patient was in hemorrhagic shock caused by massive bleeding. It is worth noting that conventional hemostatic drugs did not work. The bleeding reduced after the diagnostic curettage, and no malignant cells were found in the pathological examination. She tried to stop taking erlotinib, and bleeding stopped 3 days later. She suffered from vaginal bleeding again after erlotinib rechallenge, then bleeding stopped entirely after the ending of taking erlotinib. Erlotinib-related vaginal bleeding recurred three times (**Figure 2B**). Laboratory data were generally unremarkable except for mild anemia and low progestin level during luteal stages of the menstrual cycle (**Figure 2C**). The patient had no previous gynecological symptom and was excluded from gynecological diseases by gynecologist. She also refused to replace erlotinib owing to significant clinical benefit. The bleeding was not controlled even after the erlotinib dose was changed to 75 mg daily. The patient was then treated with tamoxifen 10 mg daily without ceasing erlotinib. Vaginal bleeding stopped 5 days after taking tamoxifen. The patient intermittently took tamoxifen due to side effects such as gastrointestinal discomfort. Vaginal bleeding did not stop completely, but the amount of bleeding was significantly decreased, which supported continuing use of erlotinib for about 20 months.

**Figure 3:**
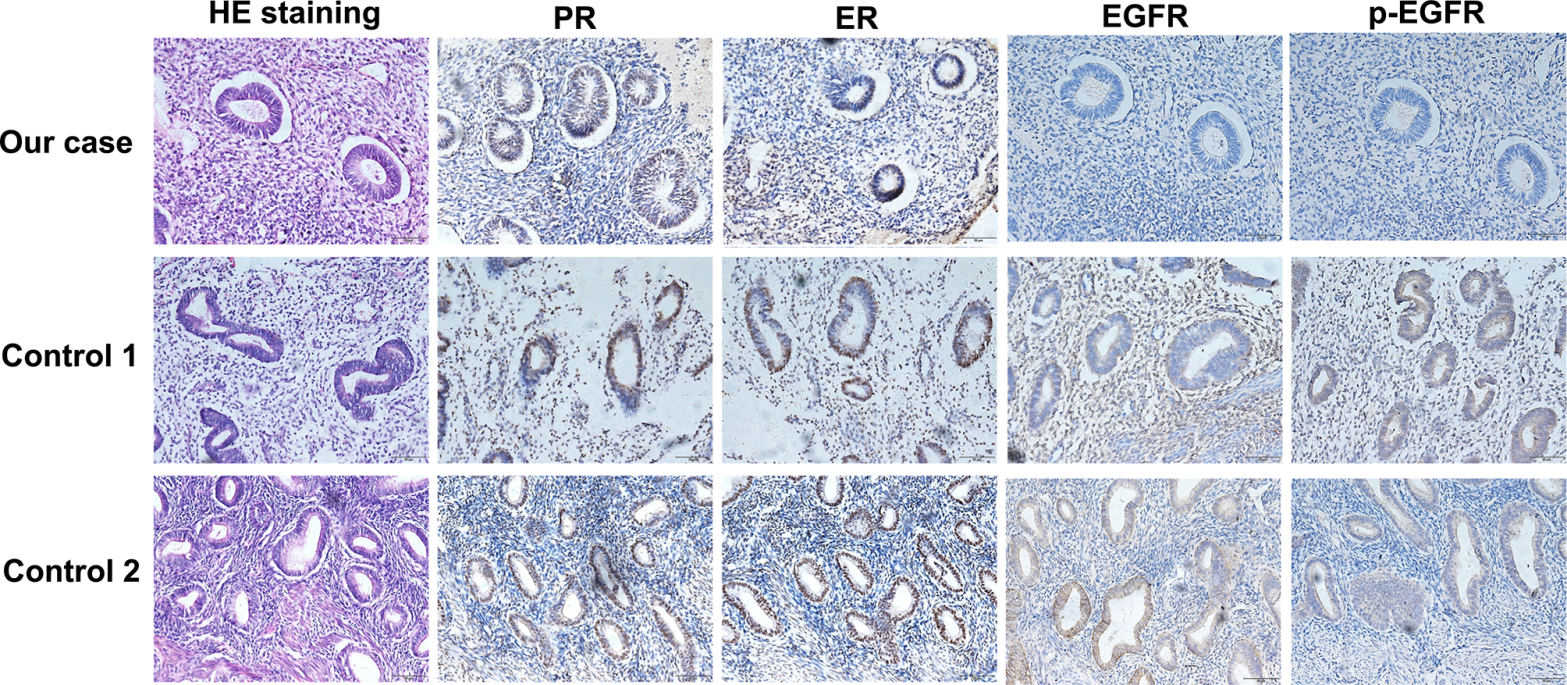
Compared with normal endometrial tissues, immunohistochemical results showed that the expression levels of EGFR and p-EGFR in endometrial tissues of the case decreased significantly.

**Figure 4:**
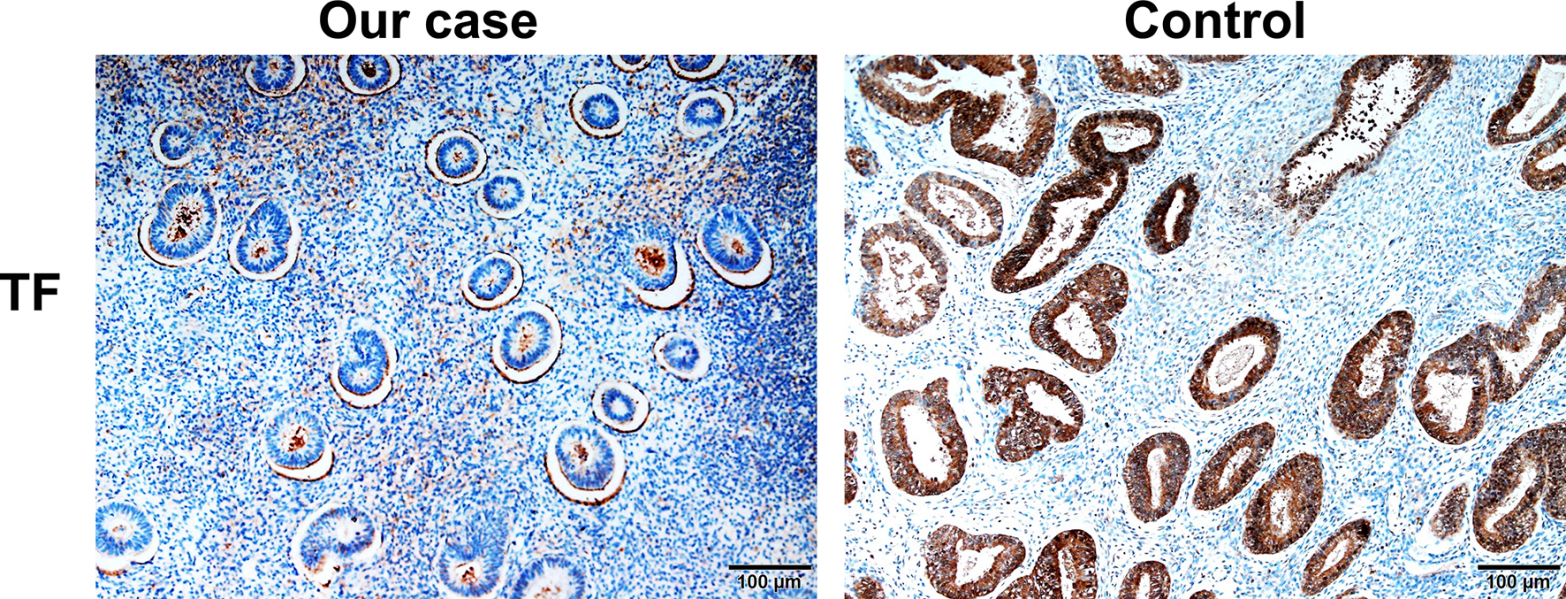
Compared with normal endometrial tissues, immunohistochemical results showed that the expression levels of TF in endometrial tissues of the case decreased significantly.

**Figure 5:**
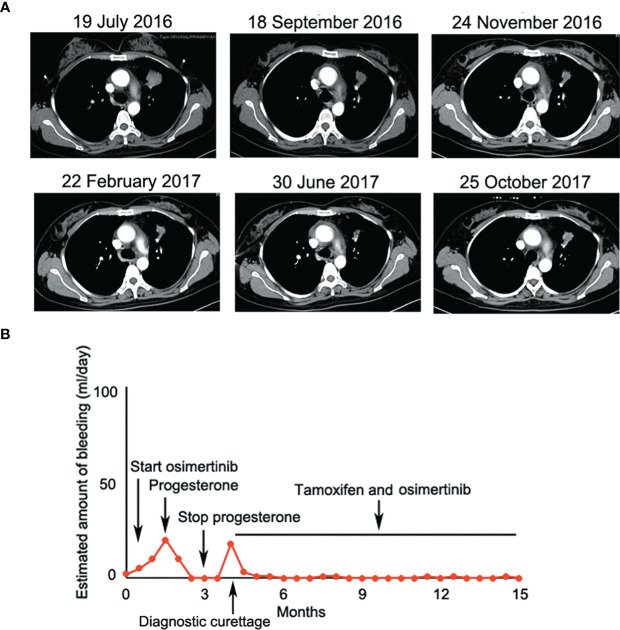
**(A)** Osimertinib decreased lesion in the upper lobe of the left lung significantly at the early stage of treatment; **(B)** Osimertinib-related vaginal bleeding during the treatment.

**Figure 6:**
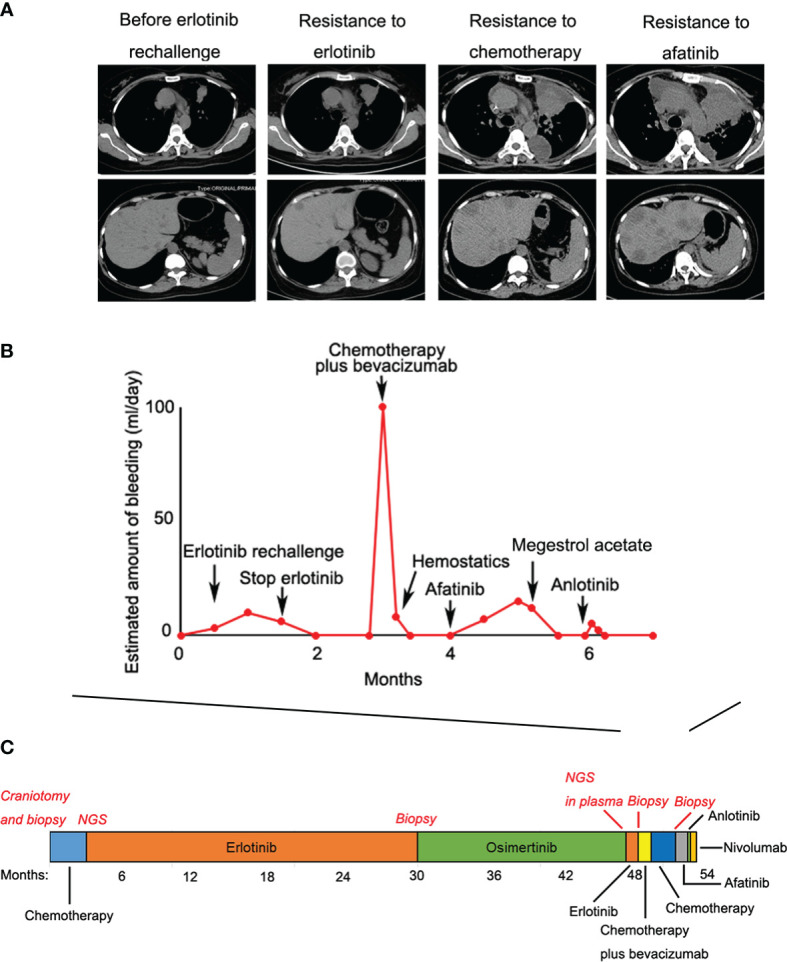
**(A)** Liver and pulmonary lesions deteriorated during the subsequent treatments; **(B)** Vaginal bleeding during the subsequent treatments; **(C)** Treatment summary and the overall survival of the patient was 53 months.

Given that erlotinib also targets wild-type EGFR, we detected EGFR expression and activation in endometrial tissues by curettage. Compared with normal endometrial tissues, immunohistochemical results showed that the expression levels of EGFR, p-EGFR and TF in endometrial tissues of the case decreased significantly (**Figures 3**, **4**). In July 2016, lung lesion was found to be progressive, and the patient was switched to 80 mg osimertinib once daily. Vaginal bleeding recurred after taking osimertinib, and bleeding completely stopped soon after subcutaneous injection of progesterone. However, the severe bleeding reoccurred when she stopped progesterone, and she had to receive another curettage. Then, she had to take tamoxifen again since osimertinib brought tumor shrinkage and vaginal bleeding again (**Figure 5**). In November 2017, progressive pulmonary lesions and new liver lesions were detected by CT scan. EGFR exon 19 deletion (2.71% mutation abundances) was found by NGS in plasma, and she tried to rechallenge with 150 mg erlotinib daily in January 2018. Vaginal bleeding occurred during erlotinib treatment, and she stopped taking erlotinib, then liver and pulmonary lesions deteriorated in February 2018 (**Figure 6A**). The patient subsequently underwent CT-guided percutaneous lung biopsy of pulmonary lesion, which revealed adenocarcinoma with EGFR exon 19 deletion without EGFR T790M and other resistant mutations. Pemetrexed/carboplatin combined with bevacizumab was administrated in March 2018. Three days after discharge, the patient developed vaginal bleeding with a volume greater than 100 mL daily. This time, the bleeding was stopped by conventional hemostatic drugs including carbazochrome sodium sulfonate (**Figure 6B**). After bleeding stopped, the patient continued to receive pemetrexed/carboplatin, but liver and lung lesions progressed after 2 cycles of chemotherapy (**Figure 6A**). The patient then took 30 mg afatinib daily, and vaginal bleeding started again soon. The bleeding stopped soon 2 days after administration of 320 mg megestrol daily. One month after administration of afatinib, progressive disease was confirmed owing to the increase of pulmonary, liver and intracranial lesions (**Figure 6A**). In June 2018, the patient received a daily 12 mg anlotinib, a novel multi-targeting tyrosine kinase inhibitor. The treatment lasted for one week, and the patient stopped the therapy due to adverse reactions such as fatigue, vomiting, and vaginal bleeding again (**Figure 6B**). Finally, she received nivolumab but did not show efficacy and died in July 2018. The overall survival of the patient was 53 months (**Figure 6C**).

## Discussion

Clinically, EGFR-TKI-induced gastrointestinal bleeding is very rare though several studies reported EGFR-TKI-induced hemorrhage such as gastrointestinal bleeding (5). However, it is not uncommon for female patients to complain of menstrual abnormalities and even prolonged vaginal bleeding. It is very important to verify whether these clinical manifestations were induced by EGFR-TKI and explore the underlying mechanism and potential treatment. We investigated the menstrual changes in premenopausal women during EGFR-TKI treatment. It indicated that 69.05% women experienced abnormal vaginal bleeding. Especially, in the reported case, the patient experienced EGFR-TKI treatment, bleeding, dosage reduction, discontinuation, rechallenge, and repeated bleeding. Nevertheless, most of female patients do not want to talk about these symptoms or can tolerate these changes, and continue to EGFR-TKI treat, resulting in both clinicians and patients to ignore this symptom. Our research suggests that we should pay attention to EGFR-TKI-induced menstruation abnormalities, notably prolonged and increased bleeding.

The mechanisms for EGFR-TKI-induced menstruation abnormalities remainunclear. In this study, the typical case provided some clues to help us to understand the underlying mechanism. In this case, it is very clear for the relationship between vaginal bleeding and EGFR-TKI including erlotinib, osimertinib and afatinib since bleeding recurred several times after the initiation of EGFR tyrosine kinase inhibitor and stopped exactly after drug withdrawal. Previous studies demonstrated that synthesis and expression of human endometrial EGF and EGFR played important role in the menstrual cycle, and EGF and EGFR levels were significantly increased at the luteal stages compared with the early follicular stage (8). TF is the primary initiator of hemostasis, which is strategically positioned to counteract the threat of hemorrhage during endovascular trophoblast invasion and subsequent induces thrombin formation. In human endometrial stromal cells, progestin increased TF mRNA and protein levels, and EGFR agonist plus progestin further enhanced TF expression (7, 9). Therefore, progestin, EGF/EGFR and TF play joint roles in menstrual cycle. The patient had low progestin level even in the luteal stages. Besides, we found that expression levels of EGFR, p-EGFR and TF in endometrial tissues of our case decreased significantly. This all indicated that low progestin and inhibition of EGFR signaling may result in decreased of TF expression. More importantly, treatment with progesterone or megestrol completely remit vaginal bleeding induced by osimertinib and afatinib. Therefore, we speculate that the downregulated EGFR signaling and low progestin level mediated decreased TF expression, which led to prolonged vaginal bleeding in our case. Progesterone or megestrol administration alleviated vaginal bleeding, this provided convincing evidence in the molecular mechanism.

We initially used tamoxifen to treat vaginal bleeding in the hope of using tamoxifen’s side effect that may induce cessation of menstruation. Besides, tamoxifen plus erlotinib resulted in cytotoxicity and cell growth inhibition synergistically in NSCLC cell lines (10). In this case, tamoxifen is effective for EGFR-TKI-induced vaginal bleeding though it does not completely solve the problem. These findings may support the combined administration of tamoxifen and EGFR inhibitor. Mechanically, however, we tend to select megestrol to combine with EGFR-TKI for better control of bleeding.

Treatment guidelines are insufficient and the optimal treatment for this uncommon drug-induced bleeding has not yet been established. As small molecular targeted therapies become an attractive therapeutic option in oncology, our study helps to recognize the unnoticeable side effects and apply the potentially effective treatment.

## Data Availability Statement

The raw data supporting the conclusions of this article will be made available by the authors, without undue reservation.

## Ethics Statement

The studies involving human participants were reviewed and approved by The Ethics Committee of West China Hospital. Written informed consent for participation was not required for this study in accordance with the national legislation and the institutional requirements.

## Author Contributions

MY and SZ collected, analyzed the patient data, and wrote the manuscript. XL, YL and YZ collected the clinical data. WW and XW performed histological examinations. YW designed the study, revised the article, and approved for the final version to be submitted. All authors read and approved the final manuscript.

## Funding

This work was supported by the National Science and Technology Major Project (2017ZX09304023) and National Natural Science Foundation (81472197) of China.

## Conflict of Interest

The authors declare that the research was conducted in the absence of any commercial or financial relationships that could be construed as a potential conflict of interest.

## Publisher’s Note

All claims expressed in this article are solely those of the authors and do not necessarily represent those of their affiliated organizations, or those of the publisher, the editors and the reviewers. Any product that may be evaluated in this article, or claim that may be made by its manufacturer, is not guaranteed or endorsed by the publisher.

## Supplementary Material

The The Supplementary Material for this article can be found online at: https://www.frontiersin.org/articles/10.3389/fonc.2022.805538/full#supplementary-material
