# Supplementary material for: Continuous Vaginal Bleeding Induced By EGFR-TKI in Premenopausal Female Patients With EGFR Mutant NSCLC
Source: Front Oncol. 2022 Jun 7;12:805538. doi: 10.3389/fonc.2022.805538 (PMC9210573; doi:10.3389/fonc.2022.805538)
Supplement: Supplementary file 1 [file Table_1.docx]

| No. | Age  (years) | Menstrual status before EGFR-TKI treatment | Menstrual status after  EGFR-TKI treatment |
| --- | --- | --- | --- |
| 1 | 49 | Normal menstruation | Scanty menstruation |
| 2 | 40 | Normal menstruation | Normal menstruation |
| 3 | 37 | Normal menstruation | Profuse menstruation |
| 4 | 45 | Normal menstruation | Normal menstruation |
| 5 | 45 | Normal menstruation | Profuse menstruation |
| 6 | 42 | Normal menstruation | Profuse menstruation |
| 7 | 32 | Normal menstruation | Profuse menstruation |
| 8 | 41 | Normal menstruation | Normal menstruation |
| 9 | 44 | Normal menstruation | Scanty menstruation |
| 10 | 45 | Normal menstruation | Scanty menstruation |
| 11 | 46 | Normal menstruation | Scanty menstruation |
| 12 | 46 | Normal menstruation | Normal menstruation |
| 13 | 31 | Normal menstruation | Normal menstruation |
| 14 | 41 | Normal menstruation | Irregular menstruation |
| 15 | 44 | Normal menstruation | Scanty menstruation |
| 16 | 43 | Normal menstruation | Scanty menstruation |
| 17 | 42 | Normal menstruation | Normal menstruation |
| 18 | 43 | Normal menstruation | Profuse menstruation |
| 19 | 41 | Normal menstruation | Scanty menstruation |
| 20 | 49 | Normal menstruation | Scanty menstruation |
| 21 | 49 | Normal menstruation | Scanty menstruation |
| 22 | 46 | Normal menstruation | Scanty menstruation |
| 23 | 42 | Normal menstruation | Profuse menstruation |
| 24 | 55 | Normal menstruation | Irregular menstruation |
| 25 | 46 | Normal menstruation | Irregular menstruation |
| 26 | 38 | Normal menstruation | Irregular menstruation |
| 27 | 49 | Normal menstruation | Irregular menstruation |
| 28 | 41 | Normal menstruation | Profuse menstruation |
| 29 | 43 | Normal menstruation | Profuse menstruation |
| 30 | 49 | Normal menstruation | Profuse menstruation |
| 31 | 52 | Normal menstruation | Profuse menstruation |
| 32 | 39 | Normal menstruation | Normal menstruation |
| 33 | 52 | Normal menstruation | Normal menstruation |
| 34 | 50 | Normal menstruation | Normal menstruation |
| 35 | 42 | Normal menstruation | Normal menstruation |
| 36 | 43 | Normal menstruation | Normal menstruation |
| 37 | 45 | Normal menstruation | Normal menstruation |
| 38 | 46 | Normal menstruation | Profuse menstruation |
| 39 | 48 | Normal menstruation | Normal menstruation |
| 40 | 46 | Normal menstruation | Irregular menstruation |
| 41 | 42 | Normal menstruation | Scanty menstruation |
| 42 | 49 | Normal menstruation | Profuse menstruation |

Supplementary table 1. The menstrual changes in 42 premenopausal women before and after EGFR-TKI treatment.
